# Antennal Morphology and Sexual Dimorphism of Antennal Sensilla in *Callitettix versicolor* (Fabricius) (Hemiptera: Cercopidae)

**DOI:** 10.3390/insects10020056

**Published:** 2019-02-19

**Authors:** Qing Zhu, Nan Wu, Jolanta Brożek, Wu Dai

**Affiliations:** 1 Key Laboratory of Plant Protection Resources and Pest Integrated Management of the Ministry of Education, College of Plant Protection, Northwest A&F University, Yangling 712100, Shaanxi, China; 18392861169@163.com (Q.Z.); wn1114691213@163.com (N.W.); 2 Department of Zoology, Faculty of Biology and Environmental Protection, University of Silesia, Bankowa 9, 40-007 Katowice, Poland; jolanta.brozek@us.edu.pl

**Keywords:** Auchenorrhyncha, antennal sensilla, fine morphology, scanning electron microscopy

## Abstract

The rice spittlebug *Callitettix versicolor* (Fabricius) is an important pest of rice and maize in South Asia and causes severe economic damage. To provide background information for chemical ecology studies, the fine morphology of antennae and the types and distribution of the sensilla on the male and female antennae of *Callitettix versicolor* (Fabricius) are investigated by means of scanning electron microscopy (SEM). Results show that the antenna is filiform and composed of three segments: a scape, a pedicel, and a flagellum. The female antennae are slightly shorter than the male antennae. In both sexes, four types and nine subtypes of sensilla were observed: sensilla basiconica (SB1, SB2), sensilla campaniformia (SCa1, SCa2 and SCa3), sensilla coeloconica (SCo1, SCo2 and SCo3) and sensilla trichodea (ST). In addition, sensilla coeloconica (SCo1) are observed on the membrane of the top of the pedicel in Cercopidae for the first time. Sexual dimorphism mainly occurs in variation in the number of sensilla coeloconica (SCo2, SCo3) on the bulb-shaped portion of the flagellum and in the shape of sensilla basiconica (SB2). There are significantly more sensilla coeloconica in males than in females. The external structure and distribution of these sensilla are compared to those of other cercopids and possible functions of the antennal sensilla are discussed.

## 1. Introduction

Members of the superfamily Cercopoidea (Hemiptera) are widely distributed, predominantly in the tropical and subtropical regions of the world, and consist of five families (Cercopidae, Aphrophoridae, Clastopteridae, Machaerotidae and Epipygidae). The family Cercopidae, known as froghoppers, includes approximately 1500 species in 150 genera [1]. Many of these species are economically significant pests of important agricultural crops. *Callitettix versicolor* (Fabricius) is one of the species harmful to agricultural crops and causes severe economic damage to rice and maize in China, India, Malaysia, Myanmar, Thailand, and Vietnam [2]. Therefore, in the study of such pest species, it is very important to know the sensory systems of their antennae, which are used for the recognition of host plants. 

Insect antennae are segmented appendages that are well-equipped with a wide variety of antennal sensilla and function primarily in chemoreception, thermoreception and hygroreception. Antennae play a crucial role in insect behavior, including host location and recognition, as well as mating behavior [3,4,5,6,7,8]. Moreover, the form of the antenna varies considerably depending on its precise function and the remarkable difference on sensory equipment, which has the potential value for taxonomic and phylogenetic analyses [9]. 

Abundant data are available on some aspects of antennae morphology of Hemiptera based on light and scanning electron microscopy [10,11,12,13,14,15,16]. Nevertheless, the antennal sensilla of cercopoid species have received only sporadic attention. Previous studies of the antennae in Cercopoidea have mostly focused upon the external comparative morphology of different cercopoid species with the aim of elucidating their taxonomic relationships [1,17] and the antennal sensilla of a few species have been studied to infer their functions [18,19]. 

Cercopoids’ antennae perform crucial functions in their life cycle, with the sensillar equipment fine-tuned by strong selection [19,20]. The clarification of the sensory mechanisms of cercopid antennae can help to understand their ecology and biology, especially where there are differences between the sexes. Some characteristics and function of the sensilla can be inferred from their morphology and anatomy [4,21]. This is best accomplished using scanning electron microscopy (SEM) techniques.

Exterior variation of the different types of sensilla and their distribution on the pedicel and flagellum in the Hemipteran infraorder Fulgoromorphan (planthoppers), have received more attention, as these structures have a potential value in taxonomic and phylogenetic analysis [9,22,23,24,25,26]. Cercopoid antennae also offer an abundance of structures that provide excellent taxonomic and phylogenetic characters [1,17]. Paladini et al. [27] reconstructed phylogenetic relationships for New World spittlebug genera based on morphological data, including a few characters of the antennae (shape of basal body of flagellum, size of basal body of flagellum relative to pedicel, length and density of setae on pedicel, arista of basal body of flagellum, and length of arista compared to pedicel) for some species (*Maxantonia rubescens, Sphenoclypeana parana, Sphenorhina rubra, Kanaima fluvialis,* and *Notozulia entreriana*) of Ischnorhininae. Moreover, there are variations in the antennal structure among genera and in some cases among species. Therefore, further study of the antennae and their sensilla will contribute to a deeper understanding of the phylogenetic relationships within the Cercopoidea [8,22,27,28,29,30,31,32]. Unfortunately, at present there is a paucity of morphological and anatomical data on antennae and antennal sensilla in species of the subfamily Cercopinae, an old and paraphyletic group. More detailed investigations with expanded taxonomic sampling are needed to address longstanding questions pertaining to generic and tribal relationships within each major spittlebug lineage [33]. 

The aim of the present study was to provide the first detailed fine morphological characterization of the antennae of *Callitettix versicolor* using light and scanning electron microscopy (SEM), because this species is a pest, and belongs to the phylogenetically problematic Old World subfamily Cercopinae. The fine structure, location and distribution of different sensilla types in both males and females were investigated to document any differences between the sexes (unusual in this group of insects), comparing the sexes and discussing the possible function of these sensilla with the aim of fully unveiling fine structural details. These data provide an important starting point and are complementary to further physiological and behavioural studies on *C. versicolor* and other cercopoid species, and may be useful for the future study of taxonomic and phylogenetic analysis of Cercopidae.

## 2. Materials and Methods

### 2.1. Insect Collecting

All *Callitettix versicolor* (Fabricius) specimens used in this study were collected in the Guizhou Province, China and preserved in 70% ethanol and stored at 4 °C.

Heads with antennae were separated from the rest of the body and rinsed in 70% ethanol in an ultrasonic cleaning. Dehydration was achieved with a graded ethanol series of 75%, 80%, 90%, and 95% each for 20 min and twice in 99.9% ethanol solutions for 30 min. The 99.9% ethanol was then substituted successively by mixing alcohol and tert-butyl ethanol with in proportions of 3:1, 1:1 and 1:3. Finally, specimens were placed in pure tert-butyl ethanol for 30 min. Specimens were dried in a freeze-drier (VFD-21S, SHINKKU VD, Tokyo, Japan) for 3 h. Thereafter, the antennae were removed from the dried samples under a stereomicroscope (Olympus SZX10, Olympus, Tokyo, Japan) and placed on aluminum stubs in various positions using double-sided sticky tape. The antennae were sputter-coated with gold-palladium (MSP-1S, Hitachi, Tokyo, Japan) and examined and observed at 15 kV in a Nova nano SEM450. Antennae of male and female adults were observed for comparison.

### 2.2. Image Processing and Morphometric Measurement

Photographs and SEMS were observed and measured after being imported into Adobe Photoshop CS6. The sensilla were classified according to their external morphology, distribution and presence or absence of pores. To characterize the sensilla, we used the nomenclature proposed by Schneider [21] and Zacharuk [3,34]. 

## 3. Results

### 3.1. Gross Morphology of Antennae

**Figure 1:**
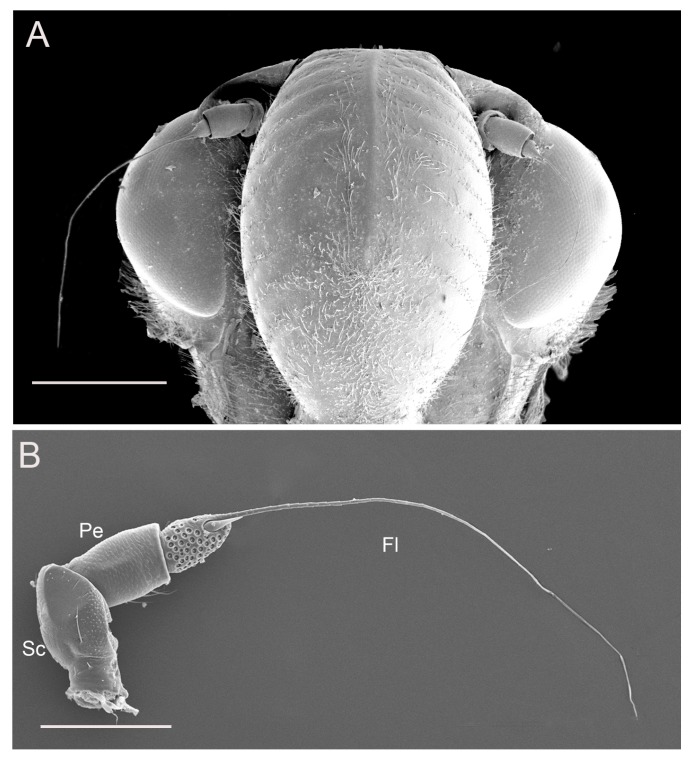
Scanning electron microscopy (SEM) images of *Callitettix versicolor* (Fabricius) female antenna. (**A**) Fronto-ventral view of the head capsule showing the insertion of the antennae and their relative position; (**B**) General view of the antenna showing the scape (Sc), the pedicel (Pe) and the flagellum (Fl). Scale bars: A = 500 μm; B = 250 μm.

The antennae are inserted in the antennal foveae on the head capsule at the side of the postclypeus and below the antennal ledges (Figure 1A). The gross morphology is similar in the male and female, consisting of three segments: a basal scape, a cylindrical pedicel and a long, filiform flagellum (Figure 1B). No significant differences were found in antennal shape between males and females. Specifically, the entire length of antennae was 1521.32 ± 10.92 μm in females (n = 5), and 1562.63 ± 11.60 μm in males (n = 5). The antennal segments were slightly longer in adult males than females, with the exception of the scape. 

**Figure 2:**
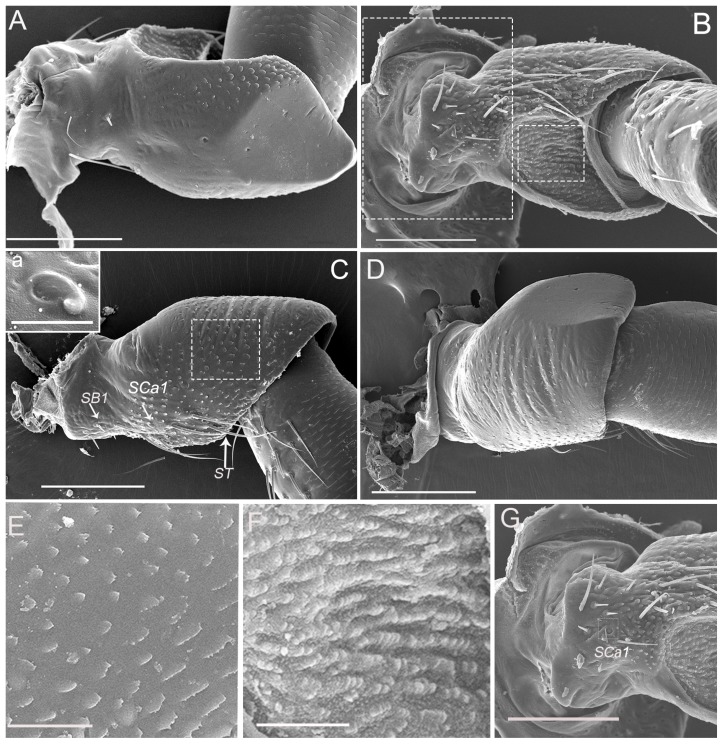
Micrographs showing the scape of an adult *Callitettix versicolor’s* (Fabricius) right antenna. (**A**) Ventral view; (**B**) Right lateral view; (**C**) Dorsal side showing sensilla trichodea (ST), sensilla basiconica (SB1) and sensilla campaniformia (SCa1); a. High magnification image of SCa1; (**D**) Left lateral view; (**E**) High magnification image of scape surface showing imbricate papillae, indicated by the rectangle given in (**C**); (**F**) High magnification image of the surface of concave on the scape, indicated by the small rectangle given in B); (**G**) High magnification image showing the antenna inserted in antennal foveae on the head capsule, the positions of the images are indicated by the big rectangle given in (**B**). Scale bars: A, B, C, D = 100 μm; E, F = 25 μm; G = 100 μm; a = 5 μm.

The scape, which connects to the antennal socket where it functions as the fulcrum for the movement of the antenna, is about 260 μm long, irregular in shape, and prominently constricted around the base (Figure 2A–D). The outer margin of the scape is conspicuously concaved in the apical half (Figure 2B); the inner margin is slightly convex, with scattered small imbricate papillae and sparsely covered with sensilla (Figure 2D). The dorsal surface is slightly convex with scattered small imbricate papillae (Figure 2C), while the ventral surface appears flat and smooth posteriorly (Figure 2A). The apex of scape is irregular in shape with the relative protrusion of the dorsal and ventral margin, which can serve a protective function by holding part of the pedicel. Therefore, the scape-pedicel joint is a ginglymus (hinge joint), indicating that the primary movement of the pedicel is in a single plane (Figure 2A,C).

**Figure 3:**
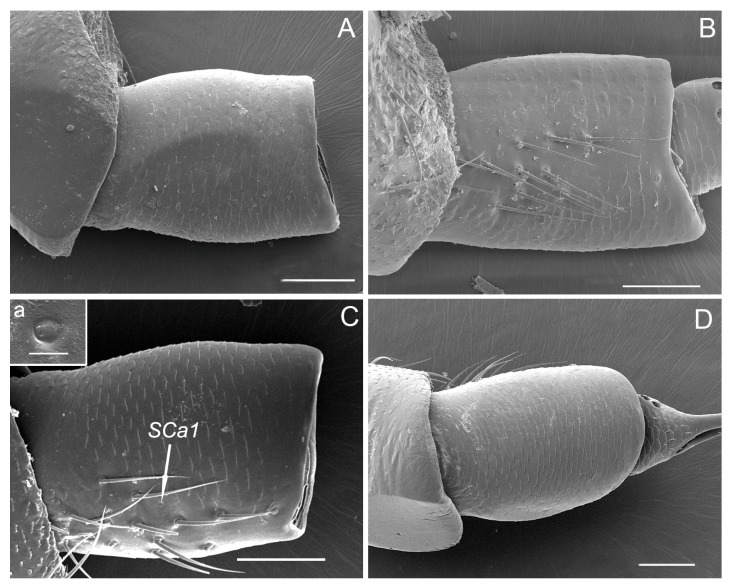
SEM micrographs showing the pedicel of adult *Callitettix versicolor* (Fabricius) right antenna. (**A**) Ventral view; (**B**) Right lateral view showing sensilla trichodea (ST); (**C**) Dorsal view, a. high magnification image of SCa1; (**D**) Left lateral view. Scale bars: A, B, C, D = 50 μm; a = 5 μm.

**Figure 4:**
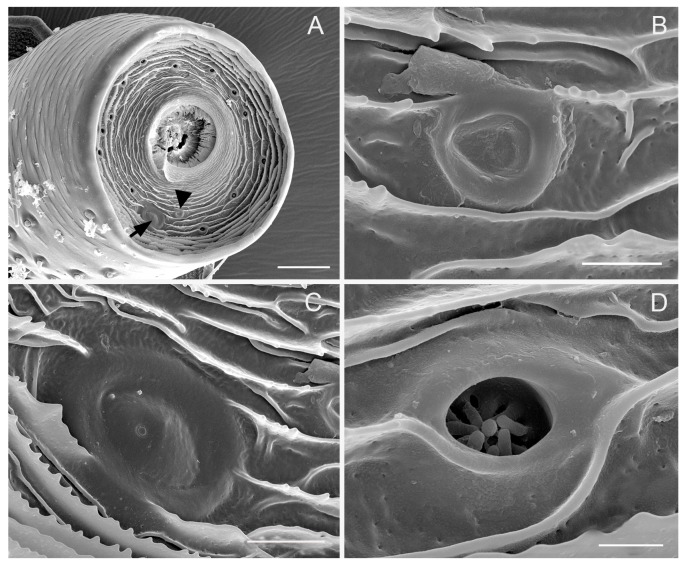
Antennal structures on the apical surface of the pedicel of adult *Callitettix versicolor* (Fabricius). (**A**) General view of the apical surface of the pedicel (black triangle indicates SCa2 and black arrow indicate SCa3), concentrically arranged cuticular ridges are visible; (**B**) High magnification image of sensilla campaniformia 1 (SCa1); (**C**) High magnification image of sensillum campaniformium 3 (SCa3); (**D**) High magnification image of sensillum coeloconicum (SCo1). Scale bars: A = 25 μm; B = 2.5 μm; C = 5 μm; D = 1.5 μm.

The pedicel, connected proximally to the concave distal end of the scape and distally to the bulb-shaped portion of the flagellum, is a short (about 200 μm) and generally cylindrical segment, slightly widened distally, wrinkled with numerous transverse small imbricate papillae and covered with several sensilla trichodea on the outer surface (Figure 3A–D). The apical concave portion of the pedicel is encircled by concentrically arranged cuticular ridges (Figure 4A). 

**Figure 5:**
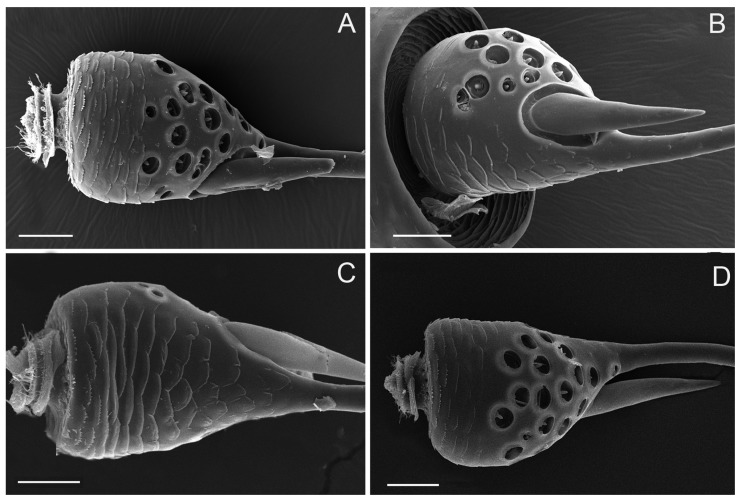
SEM micrographs showing the bulb-shaped portion of the flagellum in an adult female *Callitettix versicolor’s* (Fabricius) antenna. (**A**) Posterior side; (**B**) Ventral side; (**C**) Anterior side; (**D**) Dorsal side. Scale bars: A, B, C, D = 25 μm.

**Figure 6:**
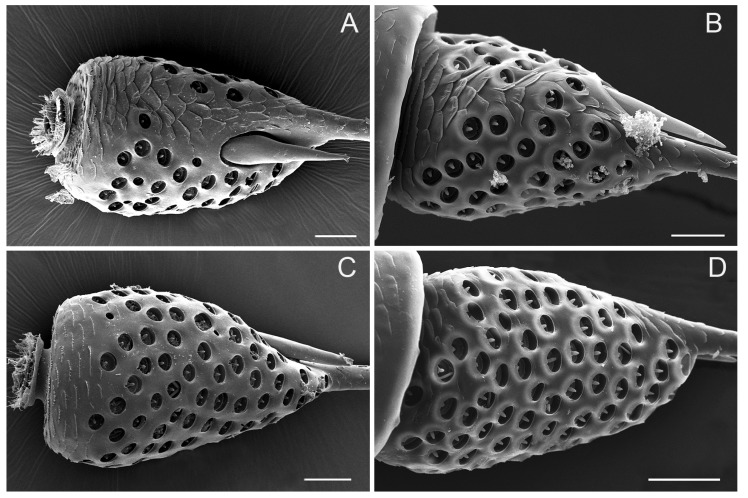
SEM micrographs showing a bulb-shaped portion of flagellum in an adult male *Callitettix versicolor’s* (Fabricius) antenna. (**A**) Ventral side; (**B**) Anterior side; (**C**) Dorsal side; (**D**) Posterior side. Scale bars: A, B, C, D = 25 μm.

**Figure 7:**
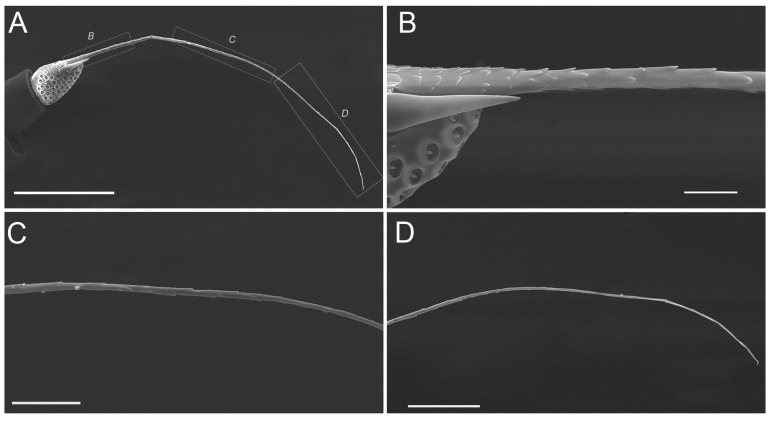
SEM micrographs showing the flagellum of an adult *Callitettix versicolor* (Fabricius). (**A**) Entire flagellum consisting of the bulb-shaped portion and apical arista; (**B**) High magnification image of the base showing apical arista covered with imbricate papillae; (**C**) High magnification image of the middle showing apical arista with imbricate papillae decreasing gradually; (**D**) High magnification image of apical half showing apical arista without imbricate papillae. Positions of images (**B**–**D**) are indicated by lettered rectangles given in (**A**). Scale bars: A = 250 μm; B = 25 μm; C = 50 μm; D = 100 μm.

The flagellum is divided into two distinct portions, an expanded basal bulb-shaped portion and a threadlike apical arista (Figure 1A,B). The bulb-shaped portion is covered with many sensory units (Figure 5A–D, and Figure 6A–D) while the threadlike arista is not segmented and is covered with imbricate papillae in half base (Figure 7A–D).

### 3.2. Sensilla Types

Four types and nine subtypes of sensilla are present: sensilla trichodea (ST) observed only on the scape and pedicel, two subtypes of sensilla basiconica (SB1, SB2) based on structure and size, three subtypes of sensilla coeloconica (SCo1, SCo2, SCo3) which differ in size and structure, and three subtypes of sensilla campaniformia (SCa1, SCa2, SCa3) based on size. 

There is apparent sexual dimorphism in antennal sensilla abundance and distribution. 

### 3.3. Sensilla Trichodea (ST)

**Figure 8:**
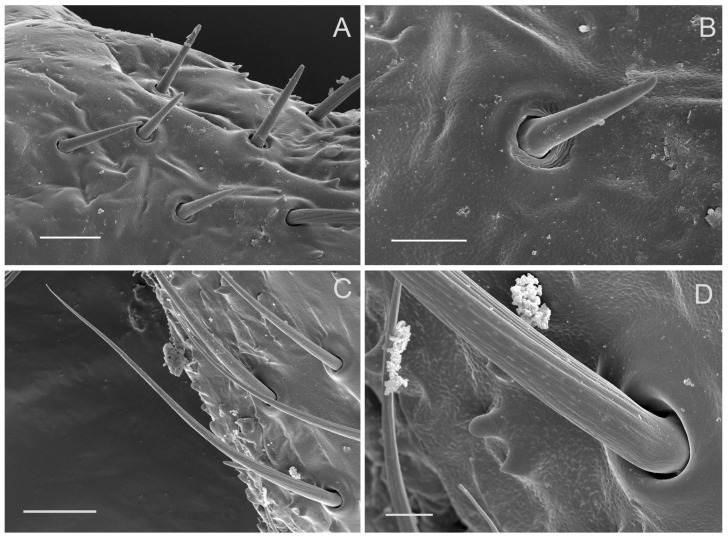
Antennal sensilla on scape and the pedicel of adult *Callitettix versicolor* (Fabricius). (**A**) Base of scape showing distribution of sensilla basiconica 1; (**B**) High magnification of sensilla basiconica 1 (SB1); (**C**) Sensilla trichodea (ST); (**D**) High magnification image of the base of sensilla trichodea (ST) showing longitudinal grooves. Scale bars: A = 10 μm; B = 5 μm; C = 15 μm; D = 2.5 μm.

Sensilla trichodea grouped into a cluster are mainly concentrated on the outer margin of the scape and pedicel (Figure 2B,C and Figure 3B,C). ST are long, straight or slightly curved toward the apex of the segment (Figure 3B,C and Figure 8C). Each sensillum trichodeum inserts in a flexible socket, which is slightly elevated above the surrounding cuticle. Each sensillum exhibits longitudinal grooves that spiral slightly and extend from the base to the remaining 2/3, and gradually fade away to the tip (Figure 2C, Figure 3B, and Figure 8C,D). The lengths of these sensilla are 56.33 ± 3.85 μm (Table 1). There are no significant differences between the male and female in length.

### 3.4. Sensilla Coeloconica (SCo)

**Figure 9:**
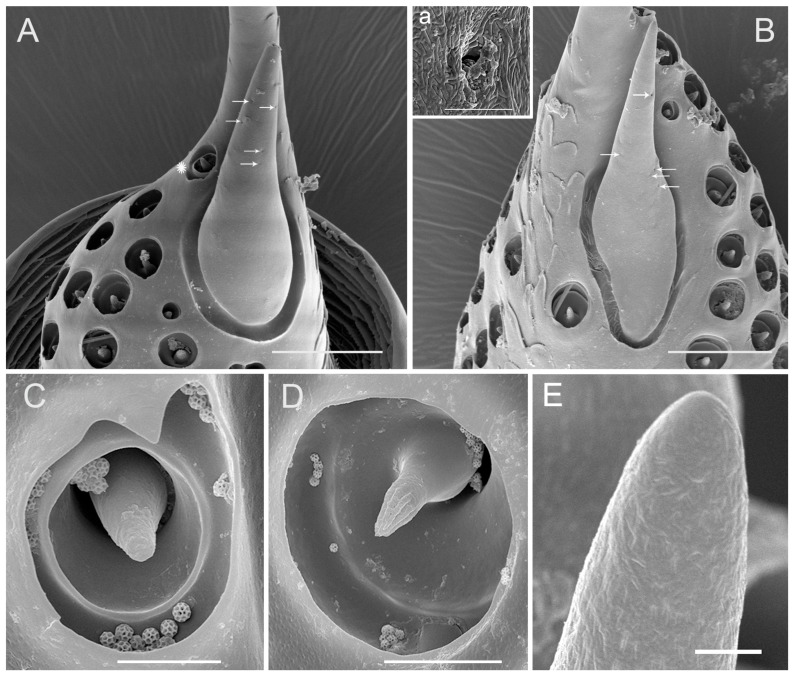
Antennal sensilla on the bulb-shaped portion of the flagellum of an adult *Callitettix versicolor* (Fabricius). (**A**) and (**B**) High magnification image of sensilla basiconica (SB2) in females and males, respectively (white stars indicate the location of SCo2 and the white arrow indicates the presence of several pitted wall pores), a. enlargement of pitted wall pore; (**C**) High magnification image of sensilla coeloconica 2 (SCo2); (**D**) High magnification image of sensilla coeloconica 3 (SCo3); (**E**) High magnification image of the apex of SB2, pores are visible. Scale bars: A = 25 μm; B = 25 μm; C = 2.5 μm; D = 5 μm; E = 1 μm; a = 0.5 μm.

Sensilla coeloconica (SCo) cover a bulb-shaped portion of the flagellum and the membrane on the top of the pedicel (Figure 4D, Figure 5A–D, and Figure 6A–D). Sensilla coeloconica are peg-in-pit sensilla, consisting of a central peg set on the bottom wall of a chamber sunken into the opening cavity (Figure 4D, and Figure 9C,D). Based on the differences in the central peg, they are divided into SCo1, SCo2 and SCo3. In SCo2, the peg is smooth and broad basally, subapically constricted and apically acute (Figure 9C). The pit has an external diameter of 4.37 ± 0.58 μm (Table 1); the base of the sensillum is inserted at the bottom of the cavity. In SCo3, the central peg is usually broad basally, subapically constricted and apically acute, with 9–12 apical groove, while the proximal half of the cuticular shaft is smooth externally (Figure 9D). The pit has an external diameter of 8.03 ± 1.02 μm (Table 1); the base of the sensillum was inserted at the bottom of the cavity. A significant difference in the quantity of sensilla between males and females was observed. In the female, there were 19–21 sensilla coeloconica including SCo2 and SCo3, located at the upper, ventromedial side of the expanded flagellar base. In males, the bulb-shaped portion was surrounded by sensilla coeloconica SCo2 and SCo3 in all directions and there were approximately 105–111 (Figure 5A–D and Figure 6A–D). Their positions may vary slightly from one individual to another. Compared with SCo2 and SCo3, SCo1 appears on the membrane of the top of the pedicel and each have an opening in which many scattered pegs are set on the bottom wall and gradually gather together (Figure 4A,D). The amount of SCo1 are approximately 13. The pit has an external diameter of 2.17 ± 0.36 μm (Table 1).

### 3.5. Sensilla Basiconica (SB)

Sensilla basiconica of two subtypes (SB1 and SB2) are observed on the antennae in both males and females. SB1 are peg-like structures with a smooth cuticle that tapers to a blunt apex and are embedded in a flexible socket (Figure 2B,C; Figure 8A,B). They occur on the base of the scape at the joints between the scape and the head. The SB1 measures 11.96 ± 1.12 μm in length. SB2 (Figure 9A,B) differs in shape and size between the sexes. SB2 are present on the bulb-shaped portion of the flagellum and are inserted in an inflexible socket on the antennal wall near the filament. SB2 in a female has a quite broad base (Figure 9A) but in a male it has a narrower base (Figure 9B). SB2 gradually tapers to an acute tip near the flagellar extension, with a depressed and pitted cuticular surface. Several wall pores are found on these sensilla basiconica 2 (Figure 9A,B), which set with their openings on the floors in the sensillar surface. Moreover, SB2 measures 57.08 ± 2.84 μm in length for female antennae and measures 72.10 ± 2.33 μm in length for male antennae (Table 1). 

### 3.6. Sensilla Campaniformia (SCa)

A sensillum campaniformium 1 (SCa1) (4.67 ± 0.56 μm in diameter) (Table 1) is situated on the dorsal surface near the sensilla trichodea of the scape and pedicel, respectively (Figure 2C, Figure 3C) and consists of a convex, button-like central area surrounded by a ring of raised cuticle (Figure 2C-a, Figure 3C-a). 

There are two sensilla campaniformia on the membrane of the top of the pedicel, which can be divided into two subtypes based on the shape and diameter (SCa2, SCa3) (Figure 4A–C). SCa2 is dome-like with the central part slightly concave, with the external diameter of 5.91 ± 0.75 μm (Figure 4B). SCa3 is similar to SCa2 in structure except for the central part being slightly convex and an ecdysial pore, which can be observed on this convex surface. The diameter of SCa3 is bigger than in SCa1 and SCa2, with an external diameter of 12.55 ± 1.01 μm (Table 1) (Figure 4C).

## 4. Discussion

In this article, we examine *Callitettix versicolor* (Fabricius), revealing the external morphology and distribution of the antennal sensilla in both males and females for the first time. The antenna sensillum types in different species of Cicadomorpha exhibit considerable structural similarity, and thus a common nomenclature can be applied. Based on these morphological observations, we find that the antennal sensilla of *C. versicolor* are similar to those of other previously studied species of the Cercopoidea [17,19]. *C. versicolor* differs in exhibiting sexual dimorphism in the number of sensilla coeloconica (SCo2 and SCo3) on the flagellum and in the shape of the sensilla basiconica. Moreover, this species has sensilla coeloconica (SCo1) on the membrane of the top of the pedicel. These have not been reported in other cercopid species, although one type of single sensilla campaniformium was reported in four species of Cercopidae [17] and a single sensillum campaniformium occurs in the Aphrophorid *Philaenus spumarius* (L.) in this part of antennae [19]. 

### 4.1. Types and Functions of Antennal Sensilla

Sensilla trichodea are the most widely distributed and abundant sensilla found on insect antennae and their occurrence has been reported for numerous Hemiptera [35,36,37,38,39]. In *C. versicolor*, sensilla trichodea are numerous, and are mainly located on the scape and pedicel, and there are no significant differences between male and female. Exteriorly, mechanoreceptive hairs in insects typically bear no pores or openings on their surface and usually possess a sharp apex and may bear cuticular sculpture such as grooves and spicules with the hair attached to a flexible socket [40]. The sensilla trichodea on the antennal scape and pedicel observed on the antennae of *C. versicolor* lack pores and are, therefore, likely to be mechanoreceptive sensilla that can perform a mechanosensillar function (tactile) [21,41,42,43].

Sensilla basiconica commonly occur in the Cercopidae and are mainly located on the bulb-shaped portion of the flagellum. Two subtypes of sensilla basiconica (SB1, SB2) were found in this study. The morphology of the SB1 on the scape in *C. versicolor* is a typical “poreless sensilla with a flexible socket” used to detect the respective antennomere position in relation to the head, thereby acting as a proprioceptor. The putative functions of the sensilla can be deduced from the number of pores, and the aporous sensilla are usually mechanoreceptors or thermo-hygroreceptors [44,45,46,47,48,49]. In contract, the morphology of SB2 in *C. versicolor* is consistent with an olfactory function, given the presence of multiple pores. Previous work on the sensilla of cercopids and other insects indicated that some sensilla basiconica are typical olfactory chemoreceptors [17,49,50,51]. There is no sexual dimorphism in the distribution and number of SB1 but sexual dimorphism in fine structure of SB2 occurs in *C. versicolor*. The shape of SB2 varies between the males and females we examined. Compared to the female, SB2 is narrower at the base in the male. Typically, in previous studies of Cercopidae and Aphrophoridae [17,19], the sensilla basiconica occurs at the apex of the bulb-shaped portion of the flagellum and their number varies between taxa, with cercopids having one and aphrophorids having three. One or four sensilla basiconica were reported in other families of Cercopoidea. 

Sensilla campaniformia are found on different parts of antennae of Auchenorrhyncha. They have been reported on the scape, near the segmental joints or the membrane of the top of the pedicel [14,17,52,53], and are considered mechanoreceptors that detect cuticular stress [3,21,40,50]. In *C. versicolor*, three subtypes of sensilla campaniformia (SCa1, SCa2 and SCa3) are located on the scape, pedicel or the membrane of the top of the pedicel. The Sca1 and Sca3 are similar to those reported in other cercopoid species: *Philaenus spumarius* (L.) (Aphrophoridae), *Aufidus trifasciatus* Stål, *Euryaulax carnifex* (Fabricius), *Petyllis deprivata* (Walker) and *Tonnoiria tasmaniae* Lallemand (Hemiptera: Cercopidae), and *Laodelphax striatellus* (Fallén) (Hemiptera: Delphacidae) [1,17,19]. Previous authors recognized only one type of sensillum campaniformium (SCa3), we reported other two types (SCa1 and Sca2). In appearance, the SCa3 in *C. versicolor* is nearly identical with the sensillum campaniformium [12] found in the cixiid *Hyalesthes obsoletus* Signoret. Generally, sensilla campaniformia are situated in areas of the cuticle that are subject to stress. The sensillum campaniformium located at the distal part of the pedicel in aphids is also considered a proprioceptor [54]. These are stimulated by mechanical deformations of the cuticle, which can either be brought about by external forces or by self-generated movements [55]. The ultrastructure of the SCa2 must be studied further to confirm this function. 

As in the present study, the literature includes reports of different numbers and distributions of sensilla coeloconica among cercopid species. For example, these sensilla are more numerous in *C. versicolor* than in *Notozulia entreriana* (Berg) [20] but the number can also vary considerably among individuals of the same species. Thus, numbers of sensilla coeloconica are generally not consistent enough to distinguish particular taxa or sexes. Sensilla coeloconica are embedded in deep open cavities and the aperture is surrounded by a ridge that can take different shapes: e.g., a single ring, petal-like wall, fringed or digitate wall. Sensilla coeloconica on the antennal flagellum of *C. versicolor* are classified into three main morphological subtypes (SCo1, SCo2 and SCo3) including digitate, petal-like or fringed walls, respectively. The first subtype of sensillum coeloconicum (SCo1) is located on the membrane of the top of the pedicel and, so far, it has not been found in other cercopid species. In the SEM, this sensillum is easily recognized due to the ‘fence’ of cuticular spines that surrounds the centrally placed sensory peg. Similar sensilla were found in many insects, however, the fence may be lacking in the distal part of the antenna [56]. This sensillum is located in the proximity of sensilla campaniformia. The function of SCo1 is not known. Further studies on the distribution of this structure (SCo1) in other species and studies to test its function are necessary. 

SCo2 and SCo3 are located on the bulb-shaped portion of the flagellum and they have a different function. According to their morphology, the Sco3 exhibits olfactory features (pores in grooves) while Sco2 has no pores and probably acts as a thermo-hygroreceptive or thermo-olfactory sensillum. SCo2 is similar to SCo3 in some other studied Cercopoidea. In Cercopidae and Aphrophoridae species, they exhibit a simple rim with a smooth margin, and are sunken below the cuticular surface of the bulb-shaped portion of the flagellum in some Australian cercopid species [17] and *P. spumarius* (L.) [19]. The SCo2 and SCo3 in *C. versicolor* are homologous to those of *P. spumarius* which consists of two different sensory pegs embedded into two separate chambers with a common, single external aperture [19]. It is likely that the SCo2 and SCo3 of *C. versicolor* play a similar function in *P. spumarius*. Ranieri et al. [19] suggested that the SCo3 of *P. spumarius* are thermo-/chemosensory receptors, with a role in olfaction. In many other insects, sensilla coeloconica on the antennae also belong to two subtypes based on the morphology and ultrastructure, and serve as olfactory sensilla when a double wall and wall pores are present, or as thermo-chemosensory sensilla when wall pores are lacking [48,49,51,57,58,59,60]

### 4.2. Morphological Characters

An essential difference among all studied cercopid species is the varied distribution of the large group of sensilla coeloconica and the shape of the sensilla basiconica. The number and shape of these sensilla vary among taxa and can be taxonomically and phylogenetically informative. This study has shown that the antennal sensilla of *C. versicolor* are quite different compared to three species of Australian cercopid species (*A. trifasciatus, P. deprivata* and *E. carnifex*), the African species *Locris maculata* [18] and the aphrophorid *Sounama borneensis* [1] in which sensilla coeloconica are concentrated in a large depressed pit. *C. versicolor* also differs from the Australian cercopid *T. tasmaniae* [17] in which eight sensilla coeloconica are arranged in two medium-sized cavities including two or three sensilla coeloconica each, and there are two separate sensilla coeloconica. However, *C. versicolor* is similar to *N. entreriana* and the aphrophorid *P. spumarius* in having the sensilla coeloconica separately located in single hollows on the bulb of the flagellum. Slight differences in the shapes of the sensilla basiconica present in most cercopid species except *N. entreriana* (long and narrow sensillum basiconicum). Significant differences are observed in the number of sensilla basiconica between the two families, with three presents in aphrophorids and usually one in most cercopids (lacking in some). Sensilla in the cercopid *Sphenoclypeana parana* (Distant) (Ischnorhininae) [27] deviate from the basic plan of other cercopids (Cercopinae), with several short sensilla basiconica and numerous sensilla coeloconica inserted in single cavities in this species. Less significant is the slight variation in number and arrangement of sensilla trichodea and campaniformia near the base of the pedicel or rarely on other areas of the antenna.

### 4.3. Sexual Dimorphism of Olfactory Sensilla

In *Callitettix versicolor,* some differences in sensilla coeloconica and basiconica (number, shape and distribution) are evident between sexes, although males and females have identical sensilla types. Significant sexual dimorphism occurs in the higher number of sensilla coeloconica/olfactory in the male than in the female. Sensilla basiconica also differ in their shape between sexes, being wider at the base and shorter with a tapered end in females in contrast to the males (SB2). This sexual dimorphism in sensilla is the first reported for a cercopoid. In related taxa of cercopids, Liang and Fletcher [17] did not observe any difference in shape and numbers of these sensilla between sexes. Sexual dimorphism was also not observed in the antennal structures and sense organs of *P. spumarius* (Aphorphoridae) [19]. In planthoppers, e.g. *L. striatellus* (Delphacidae), minor sexual dimorphism occurs in the number of sensilla (usually slightly more in the female) but not in the arrangement of sensilla types [61]. Sexual dimorphism occurs in the olfactory sensilla placodea in adults of *Lycorma delicatula* (White) (Fulgoridae); in this case with a significantly higher number and size of sensilla placodea in females than males [9]. 

The lack of sexual dimorphism in antennal sensilla in some other species of cercopids [17] offers evidence that these structures do not play a significant role in locating mates. Observations on some delphacids also suggests that antennal amputation does not influence the mating behavior of *Nilaparvata lugens* Stal [62] in which male and females communicate via substrate-borne vibrational signals transmitted and detected by organs in the abdomen [63]. According to Stacconi and Romani [61], the antennae of *L. striatellus* are unlikely to play a role in detecting mating signals but are important in olfactory orientation to the host plant [64] because numerous olfactory sensilla on the antennae of this species are used by both sexes to locate their host plants. The large number of these sensilla on the antennae of male *C. versicolor* increases the spatial density of the chemical reception, and might be useful for the detection of volatile compounds released by host plants. Alternatively, these antennal sensory structures might be related to some basic courtship behavior of the male. Chen and Liang [65] observed a self-regulatory pheromone controlling aggregation in the spittlebug nymphal stages of *C. versicolor*, suggesting that pheromones could also be used in the adult stage of this species. 

Since males of various groups of Auchenorrhyncha: Cixiidae, Flatidae, Cercopidae, Membracidae and Cicadellidae use vibrational signals to locate females, olfactory communication may be absent or at least less important in the courtship behavior of these insects [66,67,68,69,70,71,72,73,74]. Further study of *C. versicolor* will be needed to determine the behavioral or ecological significance of the observed sexual dimorphism in the antennal sensilla of this species.

## 5. Conclusions

This study provides insight into the first detailed fine morphological characterization of the antennae of *C. versicolor* and the fine structure, location and distribution of different sensilla types in both males and females. Judging by the morphology and function, the basal set of types/subtypes of the antennal sensilla of *C. versicolor* did not strongly differ from other species of Cercopidae, meaning that a similar pattern of antennal sensilla is visible. In particular, we reported other two types of sensilla campaniformia and additional type of sensillum coeloconicum. Novelty is also the sexual dimorphism in sensilla the first reported for a cercopoid. We hope this work will helpful for further physiological and behavioural studies on *C. versicolor* and other cercopoid species, and may be useful for the future study of taxonomic and phylogenetic analysis of Cercopidae. 

## Figures and Tables

**Table 1:** Morphometric data for various antennae sensilla of adult *Callitettix versicolor* (Fabricius). Data are mean ± SE values obtained from scanning electron microscopy. N = sample number; NA: not applicable.

Sensilla Type	Distribution	Length (μm)	External Diameter (μm)	N	Amount
Sensilla trichodea	Scape and pedicel	56.33 ± 3.85	2.71 ± 0.18	7	24–30
Sensilla basiconica 1	Scape	11.96 ± 1.12	2.32 ± 0.07	7	6–7
Sensilla basiconica 2	Flagellum	57.08 ± 2.84 (♀) 72.10 ± 2.33 (♂)	20.37 ± 0.99 18.49 ± 0.63	5	1
Sensilla campaniformia 1	Scape and pedicel	NA	4.67 ± 0.56	5	1
Sensilla campaniformia 2	Tip of pedicel	NA	5.91 ± 0.75	5	1
Sensilla campaniformia 3	Tip of pedicel	NA	12.55 ± 1.01	5	1
Sensilla coeloconica 1	Pedicel	NA	2.17 ± 0.36	8	13
Sensilla coeloconica 2	Flagellum	NA	4.37 ± 0.58	8(♀) 8(♂)	3–4 4–6
Sensilla coeloconica 3	Flagellum	NA	8.03 ± 1.02	8(♀) 8(♂)	16–18 100–106
